# Low birth weight and prenatal care in Colombia: a cross-sectional study

**DOI:** 10.1186/s12884-015-0541-0

**Published:** 2015-05-20

**Authors:** Ángela María Pinzón-Rondón, Vivian Gutiérrez-Pinzon, Humberto Madriñan-Navia, Jennifer Amin, Paula Aguilera-Otalvaro, Alfonso Hoyos-Martínez

**Affiliations:** Escuela de medicina y ciencias de la salud, Universidad del Rosario, Bogota, Colombia

**Keywords:** Low birth weight, Prenatal care, Social conditions, Colombia, Child health

## Abstract

**Background:**

Low birth weight (LBW) is one of the most important factors affecting child morbidity and mortality worldwide; approximately one third of neonatal deaths are attributable to it. Most research and public health policy on LBW arise from developed nations, despite that most cases (96.5%) take place in developing countries. The specific features of prenatal care that prevent LBW in developing countries are unclear. This study aims to identify the characteristics of prenatal care associated with LBW in a developing country as Colombia.

**Methods:**

Observational cross-sectional study using data from the Colombian Demographic and Health Survey 2010. A total of 10,692 children were included. Descriptive statistics were calculated, followed by bivariate regressions of LBW with all other study variables. Finally, stepwise logistic binomial regression analyses were done.

**Results:**

A LBW prevalence of 8.7% was found. Quality of prenatal care (95%CI: 0.33, 0.92; OR = 0.55), number of prenatal visits (95%CI: 0.92, 0.93; OR = 0.92), and first prenatal visits during pregnancy (95%CI: 1.02, 1.07; OR = 1.08) were associated with LBW even after controlling for all the studied variables. The health care provider conducting prenatal checkup, and insurance coverage, were not associated with LBW.

**Conclusion:**

This research provides information on the characteristics of prenatal care (quality, number of visits, and gestational age at first prenatal visit) which may strengthen LBW prevention in Colombia and possibly in countries with similar socioeconomic characteristics.

## Background

More than 20 million infants worldwide are born each year with low birth weight (LBW); this is an alarming number since it represents approximately 15.5 percent of all live born infants [1]. The World Health Organization (WHO) has defined this condition as the weight of live born infants of less than 2,500 grams, regardless of gestational age or any other etiology [2]. LBW is the result of either premature delivery or intrauterine growth restriction (IUGR), which are both influenced by genetic background, environmental exposure, behavioral patterns and access to health care [3].

One of the United Nations Millennium Development Goals (MDG) for 2015, seeks to reduce the under-five child mortality rate by 2/3 [4]. LBW has been recognized as one of the most important factors affecting child morbidity and mortality; between 28 and 30 percent of neonatal deaths are attributable to this condition [5,6]. As one of the most important predictors of child survival, LBW needs to be studied and understood in order to efficiently decrease child mortality. Moreover, since LBW has been also associated with increased morbidity later in life, [7] understanding this condition is not only relevant for early childhood but for adulthood as well.

Even though the prevalence of LBW is higher in developing countries (96.5% of all cases), most of the research on this topic is conducted in developed nations, [1] and the results from research conducted in industrialized nations are not necessarily applicable to less developed nations.

The under-five mortality rate in Colombia has improved during the last years, decreasing from 34 per 1000 live births in 1990, to 18 per 1000 live births in 2012 [8]. Yet, this rate is still far from the nation’s MDG target (11 per 1000 by 2015). Therefore, there is still much to do with.

Prenatal care is among the most important tools available to health care providers to detect time-sensitive, modifiable obstetric risk factors (infections, micronutrients deficiencies, metabolic and placental diseases) that may impact on optimal fetal development, and final birthweight. Even though the characteristics of an ideal prenatal care are still a matter of debate, in industrialized countries it is clear that ineffective practices are still part of the antenatal agenda [9-11]. Developing countries usually follow guidelines based on research conducted in industrialized nations. Although each developing country has its own characteristics, the importance of antenatal care in these countries seems to be underestimated; visits tend to be irregular and pregnant women do not tend to initiate prenatal care opportunely, nor do they comply with health recommendations [2]. According to Unicef, only half of all women worldwide receive an adequate prenatal care, and most of these women receiving adequate prenatal care are in the developed world [12]. Information on the characteristics of prenatal care leading to satisfactory obstetric outcomes in developing countries is insufficient. This research aims to identify the characteristics of prenatal care in a developing nation, Colombia, that are associated with LBW.

## Methods

A cross sectional study using data from the Colombian Demographic and Health Survey (DHS) phase-V was designed. The DHS is an international initiative that provides decisive information for policy formation and program planning. It uses nationally representative household surveys which includes data on population, nutrition and health. This data was collected from November 2009 to November 2010; respondents were selected through a multistage, stratified sampling procedure, including rural and urban areas [13].

### Population

The population was children born in Colombia from November 2006 to November 2010.

### Inclusion and exclusion criteria

We included all children of women, 13 to 49 years of age, who answered the Colombian DHS 2010. We excluded children without reliable information on birth weight, and children born overseas.

### Sample

The initial sample used in this study was all children born from women included in the DHS survey (13 to 49 years old) within the 36 months preceding data collection. This sample included 17,443 children, and it was reduced to 17,441 when children born overseas were excluded. The sample was further limited to those whose birth weight was either registered in the birth certificate, or the mother affirmed (during data collection) to accurately recall the birth weight and she provided a valid value. The final sample was 10,692 children.

### Variables

The outcome variable was LBW, defined as the weight at birth of less than 2,500 grams (birth weight ≥ 2,500 g = 0; birth weight < 2,500 g = 1). Birth weight was the weight of the newborn in grams at the moment of birth regardless of gestational age or any other factor that might have an influence on it. Birth weight information was gathered either from birth certificates, or from information provided by a mother who affirmed recalling the birth weight accurately and provided a valid value.

The exposure variables were the characteristics of the prenatal care. *Provider*: divided in three dummy variables −1) No antenatal care, 2) Physician and 3) Other health professional-. *Quality of prenatal care*: this variable summarizes information on the interventions that the mother received during the prenatal care: education, physical examination –weight, height, uterine height, blood pressure-, immunization –tetanus-, tests -blood and urine-, and nutritional supplements –iron and folic acid-. A factor analysis indicated that the multiple interventions represented a single concept, hence, a new scale of quality of prenatal care was created (the scale had a score from 0 to 1, 0 being lowest quality and 1 being highest quality of prenatal care, alpha Cronbach 0.898. Women with no prenatal care had a score of 0). *Number of prenatal care visits*, *First prenatal care* (month in which the prenatal care was started. For women with no prenatal care it was coded as 9), and *Health insurance coverage*: mother’s health insurance covered prenatal care (0 = no; 1 = yes).

The control variables were divided in pregnancy characteristics, child characteristics, mother characteristics and context characteristics. The following pregnancy characteristics were considered: *weeks of gestational age at delivery*, *preceding birth interval ≤ 1 year* (0 = yes; 1 = no), *substance abuse during pregnancy* (score from 0 to 3 of the amount and type of substance abuse during pregnancy -tobacco, alcohol, marijuana, heroin and cocaine-), *wanted child* (regression score from a factor analysis of three variables: wanted the child, wanted the pregnancy and when did she want to become pregnant).

The child characteristics included in the analysis were *sex* (0 = male; 1 = female) and *product of a multiple pregnancy* (0 = no; 1 = yes).

The assessed mother characteristics were *age* (in years), *education* (in single years), *employment* (the mother is employed 0 = no; 1 = yes), *marital status* (divided in three dummy variables: 1) never married, 2) currently married and 3) previously married), *height* (in centimeters), body mass index (*BMI*) (divided in three dummy variables: 1) low –less than 18.5-, 2) regular –from 18.5 to 30-, and 3) high –above 30-), *total children ever born*, *previously terminated pregnancy* (0 = no; 1 = yes), *history of sexual abuse* (0 = no; 1 = yes), *history of physical abuse* (0 = no; 1 = yes), *history of psychological abuse* (0 = no; 1 = yes), and *final say* (regression score from a factor analysis of nine variables evaluating the women’s participation on household decisions).

Finally, the following context characteristics were considered: *number of household members*, *wealth index* (calculated by the DHS, with higher grades indicating greater wealth), *urban residency* (0 = no; 1 = yes), *migration* (living in the city of residence for less than a year), *region* (divided in 6 dummy variables: 1) Atlantic, 2) Oriental, 3) Central, 4) Pacific, 5) Bogota and 6) National Territories), *household head* (divided in three dummy variables: 1) woman, 2) partner and 3) other), and sanitation (score of clean water supply and healthy indoor environment).

### Statistical analysis

Analysis of data was done using the IBM Statistical Package for the Social Sciences version 20 (IBM SPSS 20.0). A cut off P value of <0.05 was considered of statistical significance. First, descriptive statistics of all study variables were calculated -proportions for categorical variables, and mean, standard deviation, minimum and maximum for numerical variables-. Second, bivariate regressions of all study variables with LBW were obtained using chi square for categorical variables and independent sample t-test for numerical variables. Third, a stepwise binomial logistic regression of LBW on prenatal characteristics was conducted adjusting by child, mother and context characteristics. Bogota was excluded of the analysis for its collinearity with urban residence. Finally, a sensitivity analysis was conducted considering the missing values of low birthweight as low birthweight children and then considering the missing values as normal birthweight children.

### Ethical consideration

The study was conducted using secondary sources. No identifying information was available to the researchers, nor informed consent was needed. It was approved by Universidad Del Rosario School of Medicine and Health Sciences, Ethics in Research Committee.

## Results

### Descriptive statistics

The final sample included 10,692 Colombian children. The descriptive statistics and bivariate analysis of the studied variables are shown in Table 1*,* for the total population, the normal birth weight population and the low birth weight population.

**Table 1:** **Descriptive statistics and bivariate regressions of studied variables by birthweight**

**Variables**	**Min**	**Max**	**Total population n = 10,692**	**Normal birth weight n = 9,523**	**Low birth weight n = 1,169**	**P Value**
Low birth weight	0	1	10.93%			
Prenatal care						
Provider						
No antenatal care	0	1	2.41%	2.26%	3.68%	0.003
Physician	0	1	92.95%	93.74%	91.96%	0.020
Other	0	1	4.64%	4.00%	4.36%	0.634
Quality of prenatal care	0	1	M = 0.90 SD = 0.17	M = 0.90 SD = 0.17	M = 0.87 SD = 0.20	<0.001
Number of prenatal care visits	0	20	M = 6.72 SD = 2.81	M = 6.79 SD = 2.77	M = 6.12 SD = 3.08	<0.001
Gestational age at first prenatal care	0	9	M = 2.47 SD = 1.54	M = 2.46 SD = 1.50	M = 2.53 SD = 1.52	0.144
Health insurance coverage	0	1	88.10%	88.45%	85.29%	0.002
Pregnancy characteristics						
Gestational age at delivery	20	40	M = 38.82 SD = 1.99	M = 39.08 SD = 1.50	M = 36.62 SD = 3.48	<0.001
Preceding birth interval ≤ 1 year	0	1	0.27%	0.23%	0.60%	0.023
Substance abuse	0	4	M = 0.11 SD = 0.40	M = 0.10 SD = 0.40	M = 0.10 SD = 0.34	0.541
Wanted child	−2	9	M = 0.00 SD = 1.00	M = 0.00 SD = 1.00	M = −0.05 SD = 0.97	0.049
Child characteristics						
Product of a Multiple pregnancy	0	1	0.78%	0.32%	4.53%	<0.001
Sex	0	1	48.14%	47.44%	53.81%	<0.001
Mother characteristics						
Age (years)	13	49	M = 27.57 SD = 6.87	M = 27.6 SD = 6.8	M = 27.34 SD = 7.21	0.231
Education	0	23	M = 9.17 SD = 3.77	M = 9.19 SD = 3.70	M = 9.00 SD = 3.70	0.095
Employment	0	1	48.23%	48.26%	47.99%	0.860
Marital Status						
Never married	0	1	11.39%	11.18%	13.09%	0.053
Currently married	0	1	73.73%	73.91%	72.28%	0.235
Previously married	0	1	14.88%	14.91%	14.63%	0.797
Height (cm)	1066	1835	M = 1556.82 SD = 58.83	M = 1558.27 SD = 58.68	M = 1544.91 SD = 59.48	<0.001
BMI						
Low	0	1	9.20%	8.76%	12.83%	<0.001
Regular	0	1	73.93%	74.20%	71.77%	0.074
High	0	1	16.91%	17.10%	15.40%	0.144
Total children ever born	1	14	M = 2.19 SD = 1.49	M = 2.19 SD = 1.51	M = 2.16 SD = 1.51	0.425
Previously terminated pregnancy	0	1	22.46%	22.46%	22.41%	0.970
History of sexual abuse	0	1	19.09%	18.75%	21.81%	0.012
History of psychological abuse	0	1	48.13%	47.83%	50.56%	0.078
History of physical abuse	0	1	17.87%	17.91%	17.54%	0.750
Final say	0	4	M = 0.00 SD = 1.00	M = 0.00 SD = 1.00	M = 0.03 SD = 1.03	0.269
Context characteristics						
Number of household members	1	19	M = 5.25 SD = 2.23	M = 5.24 SD = 2.28	M = 5.34 SD = 2.33	0.156
Wealth index	1	5	M = 2.54 SD = 1.27	M = 2.54 SD = 1.37	M = 2.49 SD = 1.27	0.192
Region						
Atlantic	0	1	19.67%	19.61%	20.19%	0.636
Oriental	0	1	16.98%	17.14%	15.65%	0.202
Central	0	1	23.34%	23.71%	20.36%	0.011
Pacific	0	1	12.42%	12.28%	13.60%	0.195
Bogota	0	1	7.23%	6.93%	9.67%	0.001
National territories	0	1	20.36%	20.34%	20.53%	0.879
Urban residency	0	1	71.56%	71.66%	70.74%	0.513
Migration	0	1	12.03%	11.91	13.55%	0.047
Household head						
Woman	0	1	13.81%	13.67%	14.97%	0.225
Partner	0	1	53.79%	54.27%	49.87%	0.004
Other	0	1	32.40%	32.06%	35.16%	0.033
Sanitation	0	2	M = 1.04 SD = 0.46	M = 1.03 SD = 0.47	M = 1.04 SD = 0.48	0.891

P: P value; M: mean; SD: standard deviation.

A LBW prevalence of 8.7 percent was found. In the total studied population, only a small percentage of women did not attend prenatal care (2.41%). Among normal birth weight (2.26 %) and LBW (3.68%) did not attend prenatal care. On the other hand, among those who received prenatal care the vast majority received prenatal care from physicians (92.95%). The average quality of prenatal care provided was very high (0.90 in a scale from 0 to 1; in which 1 is the highest quality SD = 0.17). Women attended on average 6.72 ± 2.81 prenatal visits, with a mean gestational age for the first prenatal visit of 2.5 months ±1.54, and most women had health insurance coverage (88.10%). Deliveries occurred on average at 38.82 weeks of pregnancy ± 1.99, a very small percentage of women had a previous pregnancy less than 1 year before (0.27%), and the substance abuse was uncommon. Multiple pregnancies occurred in less than 1 percent of the cases (0.78%), and 48.14 percent of the women delivered female newborns. The mean maternal age at time of delivery was 27.57 years ± 6.87, mothers had on average 9.17 years of education ±3.77, around half of the mothers were employed (48.23%), and most of them were currently married (73.73%). The average mothers’ height was 156 cm ± 58.83, meanwhile, percentages of low, normal and high BMI were 9.2, 73.93 and 22.49 percent, respectively. In terms of abuse, approximately half (48.13%) of the mothers reported being victims of psychological abuse, close to one in five of them had been victims of physical (17.87%) or sexual abuse (19.09%), and 22.49% had had an abortion.

A statistically significant difference on the absence of prenatal care between LBW infants (3.68%) and normal birth weight infants (2.26%), was found. On prenatal care characteristics: a physician provider was most commonly found for the group of normal birth weight (93.74%), as compared to the group of LBW, quality of prenatal care was higher for the group of normal birth weight (0.90 vs. 0.87), as well as number of antenatal care visits (6.12 vs. 6.79).Mothers with normal weight newborns were more likely to have health insurance coverage for antenatal care and delivery (88% vs. 85%). Time of first prenatal visit was not associated with LBW.

In terms of pregnancy characteristics: as expected, LBW newborns were product of pregnancies with lower gestational age (36 weeks vs. 39 weeks). Infants with LBW were more likely to be the outcome of pregnancies with shorter interval of time between pregnancies (0.60% vs. 0.23%), and these infants were less likely to be wanted, as compared with those infants who had a normal birth weight.

With regard to child characteristics: pregnancies resulting in LBW were more likely to have a female newborn (53.81% vs. 47.44%) and to be a multiple pregnancy (4.53% vs. 0.32%).

Mothers of LBW infants were more likely to be of a shorter height, have a low BMI and report a history of sexual abuse (not necessarily resulting on the studied child’s pregnancy).

Finally, regarding context characteristics, mothers living in the Central region (mostly rural area) were less likely to have children with LBW, while mothers living in Bogota (Colombia’s capital and most densely populated city) were more likely to have a LBW infant. Additionally, households headed by the woman’s partner were less likely to have babies with LBW.

### Multivariate regressions

The results of the binomial logistic regressions of LBW on prenatal care variables are presented in Table 2*;* model 1 unadjusted and model 2 adjusted for all the study variables. After adjusting for all the study variables; the absence of prenatal care (OR = 2.20), the quality (OR = 0.55) and quantity (OR = 0.93) of it, as well as the gestational age at first prenatal care visit (OR = 1.08), were associated with LBW.

**Table 2:** **Binomial logistic regressions of LBW on prenatal care variables**

**Variables**	**OR**	**Sig.**	**OR**	**Sig.**
Prenatal Care characteristics	Model 1		Model 2	
Provider: No antenatal care	2.941	0.007	2.198	0.011
Provider: Physician	*Reference*			
Provider: Other	1.061	0.696	1.053	0.756
Quality of prenatal care	0.642	0.076	0.552	0.033
Number of prenatal care visits	0.864	<0.001	0.921	<0.001
First prenatal care	1.133	<0.001	1.080	0.045
Health insurance coverage	0.817	0.044	0.871	0.214
Pregnancy characteristics				
Gestational age at delivery (weeks)			0.631	<0.001
Preceding birth interval ≤ 1 year			1.214	0.690
Substance abuse			0.841	0.089
Wanted child			0.970	0.412
Child characteristics				
Product of a Multiple pregnancy			9.722	<0.001
Sex			1.402	<0.001
Mother characteristics				
Age			1.011	0.157
Education			0.962	0.002
Employment			1.063	0.421
Marital Status: Never married			0.932	0.601
Marital Status: Currently married			*Reference*	
Marital Status: Previously married			0.871	0.179
Height (cm)			0.995	<0.001
BMI: Low			1.645	<0.001
BMI: Regular			*Reference*	
BMI: High			0.886	0.226
Total children ever born			0.893	0.002
Previously terminated pregnancy			0.962	0.635
History of sexual abuse			1.152	0.009
History of psychological abuse			0.971	0.684
History of physical abuse			0.985	0.857
Final say			0.975	0.427
Context characteristics				
Number of household members			0.996	0.865
Wealth index			0.979	0.645
Urban residency			0.971	0.761
Migration			1.151	0.258
Household head: Woman			1.249	0.051
Household head: Partner			*Reference*	
Household head: Other			1.328	0.024
Sanitation			0.915	0.423
−2 Log likelihood	7274.10		2185.23	
Cox & Snell R Square	0.01		0.21	
Nagelkerke R Square	0.02		0. 44	

OR: odds ratio; Sig.: significance.

Other associated factors were weeks of gestational age at delivery (OR = 0.63), product of a multiple pregnancy (OR = 9.72), sex (OR = 1.40), maternal education (OR = 0.96), maternal height (OR = 0.95), maternal low BMI (OR = 1.65), as compared to a regular BMI, history of sexual abuse (OR = 1.15) and other household head, as compared with woman’s partner head (OR = 1.33).

#### Sensitivity analysis

In the sensitivity analysis conducted considering the low birthweight missing values as children with low birth weight, only number of prenatal care visits lost significance, while in the sensitivity analysis considering the low birth weight missing values as children with normal birthweight, there were no changes in association.

## Discussion

The prevalence found, 8.7 percent, confirms what has been reported by the Colombian National Department of Statistics (DANE) and by non-governmental organizations in the country –Asi vamos en salud-, which estimated a national LBW prevalence between 8 and 9 percent [14,15].

The absence of prenatal care, as reported by the literature (ORs between 1.85 and 2.59), [2,16-18] was associated with LBW in this paper (OR = 2.20). The provider of the prenatal care was not associated with LBW; it seems there is no difference between medical doctors and other health care providers in terms of final birthweight. The literature has reported that nurses, in particular those trained on obstetric care, are efficient to provide antenatal care [19,20]. This study confirms these findings.

Quality of prenatal care was not associated with LBW in the unadjusted model but became the factor with greater association in the adjusted one (OR = 0.55). When pregnancy, child, mother and context characteristics are taken into account, a complete prenatal care (education, physical examination, tests and immunizations) becomes essential to ensure a good pregnancy outcome, as reported by other authors [21].

The number of prenatal visits and the gestational age for the first antenatal visit were protective factors against LBW in both the unadjusted and adjusted models. The WHO recommends at least four prenatal visits and the first visit should take place during the first trimester [22]. This study provides evidence of the importance of an early start of prenatal care and the benefits of sufficient quantity of visits.

Finally, health insurance coverage showed association with LBW in the unadjusted models but not in the adjusted one.

Other factors that were associated with LBW are well documented in the literature; these factors are: preterm delivery (weeks of gestational age at delivery), [23] product of a multiple pregnancy, [24,25] female newborn, [26-28] low education of the mother, [29] maternal low height and BMI, [30] and history of sexual abuse [31].

Although this study offers some advantages such as the large sample size and quality of data , it also has important limitations. First, there are many no measured confounders such as genetics, maternal history of disease and pregnancy, among others. Second, it is based on maternal information; hence, it is subject to recall bias. Third, the available data limited the possibility to study details of the antenatal care characteristics. Fourth, approximately a third of the sample was lost because the birth weight data was missing or it was not accurately recalled. Nevertheless, after performing a sensitivity analysis the results remained very similar to those originally reported in this study, so there is no reason to believe that the sample may change the results in any particular direction. Fifth, its cross-sectional design does not allow to infer causality, although there are not reasons to believe that there are temporal ambiguities, and finally, the large sample size contributes to an over-power analysis that could detect minimal effect sizes, and these effect sizes could be the result from slight biases in the sampling process.

## Conclusion

A complete prenatal care, beginning as early as possible and including a good number of prenatal visits, appears to be of great importance to decrease LBW, and to avoid future associated complications. . On the other hand, the provision of care by a physician does not seem to be necessary to decrease LBW, as long as another health professional provides the prenatal care.

## Abbreviations

LBW: Low birth weight
DHS: Demographic and health survey
WHO: World health organization
IUGR: Intrauterine growth restriction
MDG: Millennium development goals
BMI: Body mass index
